# 1630. Clinical Profile of Acute Respiratory Illness (ARI) Events in the Phase 3 Trial The RSV Vaccine Efficacy Study iN Older Adults Immunized against RSV Disease (RENOIR)

**DOI:** 10.1093/ofid/ofad500.1464

**Published:** 2023-11-27

**Authors:** Gonzalo Pérez Marc, Edward E Walsh, Elliot N DeHaan, Conrado J Llapur, Agnieszka Zareba, Qin Jiang, Kumar Ilangovan, John Woodside, Daniel P Eiras, Tarek Mikati, Elena Kalinina, David Cooper, Annaliesa S Anderson, Kena A Swanson, William C Gruber, Alejandra C Gurtman, Beate Schmoele-Thoma

**Affiliations:** Hospital Militar Central de Buenos Aires, Ciudad Autónoma de Buenos Aires, Buenos Aires, Argentina; University of Rochester, Rochester, NY; Pfizer, Pearly River, New York; Hospital del Niño Jesús, San Miguel de Tucuman, Tucuman, Argentina; Pfizer, Pearly River, New York; Pfizer, Pearly River, New York; Pfizer, Vaccine Research and Development, Raleigh, North Carolina; Pfizer, Pearly River, New York; Pfizer, Inc., Pearl River, New York; 3. Pfizer, Inc., Vaccine Research & Development, Pearl River, New York; Pfizer, Pearly River, New York; Pfizer, Pearly River, New York; Pfizer, Pearly River, New York; Pfizer, Pearly River, New York; Pfizer, Pearly River, New York; Pfizer, Pearly River, New York; Pfizer, Pearly River, New York

## Abstract

**Background:**

RENOIR is a phase 3 randomized, double-blinded, placebo-controlled study evaluating Vaccine Efficacy (VE) to prevent lower respiratory tract illness (LRTI) in adults ≥ 60 years of age during 2 RSV seasons in Northern and Southern Hemisphere countries (NCT05035212). End of RSV Season 1 (EoS1) analysis demonstrated VE of 88.9% for RSV-associated Lower Respiratory Tract Illness (LRTI-RSV) with 3+ symptoms, VE of 65.1% for LRTI-RSV with 2+ symptoms, and VE of 62.2% for all Acute Respiratory Illness (ARI)-RSV events including LRTI-RSV 2+ and 3+ events. We sought to understand the ARI symptom distribution (e.g. upper respiratory versus lower) among all RSV confirmed endpoints, and the characterization of medical diagnoses provided for these endpoints by clinicians.

**Methods:**

As per study protocol, ARI is an illness involving 1 or more of 7 respiratory illness symptoms lasting more than 1 day: new/increased sore throat, cough, nasal congestion, nasal discharge, wheezing, sputum production, shortness of breath during the RSV season. Participants with ARI symptoms obtain a nasal self-swab within 7 days of onset for RT-PCR testing and undergo virtual or in-person evaluation. Clinical diagnoses are collected at medically attended visits or by investigator assessment. Additional Study Definitions: LRTI is an ARI with at least 2 or 3 signs/symptoms: new or increased cough, wheezing, sputum production, shortness of breath, tachypnea lasting more than 1 day. ARI-RSV and LRTI RSV is an RT-PCR confirmed ARI or LRTI.

**Results:**

At EoS1, respiratory symptoms trended lower for cough, sputum production, wheezing, shortness of breath, and tachypnea in RSVpreF recipients compared to placebo recipients. Of these wheezing, shortness of breath, and tachypnea are considered as more severe symptoms. Diagnoses of bronchitis, Influenza-like illness, and pneumonia were less common in RSVpreF recipients compared to placebo recipients.

**Figure:**
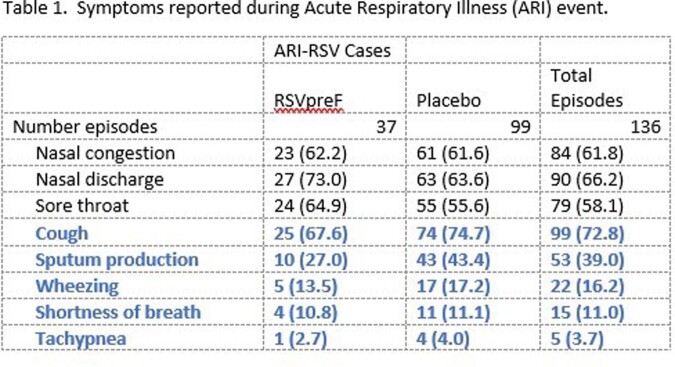

**Figure:**
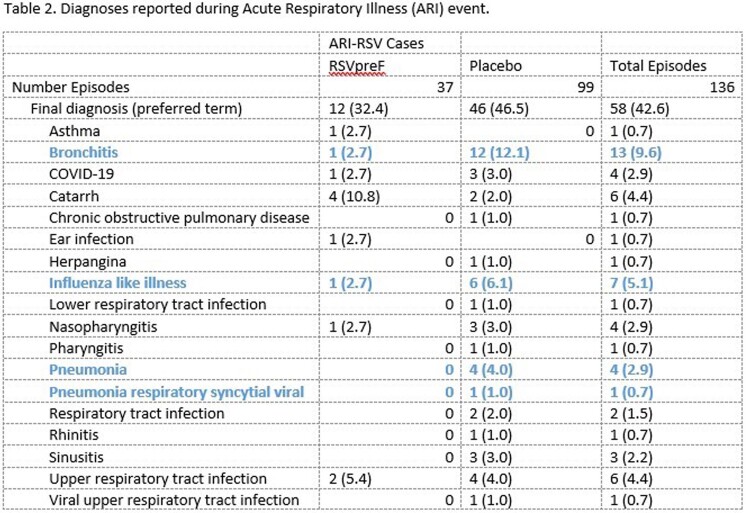

**Conclusion:**

The reduction of more severe LRTI-RSV symptoms and diagnoses amongst all ARI-RSV events in vaccine recipients reflects the higher VE against more severe RSV disease observed in the RENOIR study. The vaccine impacts symptoms that are associated with lower respiratory involvement.

**Disclosures:**

**Gonzalo Pérez Marc, M.D.**, GSK: Grant/Research Support|Merck: Grant/Research Support|Moderna: Expert Testimony|Moderna: Grant/Research Support|Pfizer: Grant/Research Support **Edward E. Walsh, MD**, Icosavax: Advisor/Consultant|Merck: Advisor/Consultant|Merck: Grant/Research Support|Merck: Honoraria|Moderna: Advisor/Consultant|Pfizer: Grant/Research Support **Elliot N. DeHaan, MD**, Pfizer: Employee|Pfizer: Stocks/Bonds **Agnieszka Zareba, MD PhD**, Pfizer: Employee|Pfizer: Stocks/Bonds|Pfizer: Stocks/Bonds **Qin Jiang, PhD**, Pfizer: Employee|Pfizer: Employee|Pfizer: Stocks/Bonds|Pfizer: Stocks/Bonds **Kumar Ilangovan, MD, MSPH, MMCi**, Pfizer, Inc.: Employee|Pfizer, Inc.: Stocks/Bonds **Daniel P. Eiras, MD, MPH**, Pfizer, Inc.: Stocks/Bonds **Tarek Mikati, MD,MPH**, Pfizer: Stocks/Bonds **Elena Kalinina, PhD**, Pfizer: Pfizer employee|Pfizer: Stocks/Bonds **David Cooper, PhD**, Pfizer, Inc.: Stocks/Bonds **Annaliesa S. Anderson, PhD**, Pfizer: Employee|Pfizer: Stocks/Bonds **Kena A. Swanson, Ph.D.**, Pfizer: Employee|Pfizer: Stocks/Bonds **William C. Gruber, MD**, Pfizer, Inc.: Employee|Pfizer, Inc.: Stocks/Bonds **Alejandra C. Gurtman, M.D.**, Pfizer: Employee|Pfizer: Stocks/Bonds **Beate Schmoele-Thoma, MD**, Pfizer: Stocks/Bonds

